# Is visual estimation of passive range of motion in the pediatric lower limb valid and reliable

**DOI:** 10.1186/1471-2474-10-126

**Published:** 2009-10-12

**Authors:** Rami Rachkidi, Ismat Ghanem, Ibrahim Kalouche, Samer El Hage, Fernand Dagher, Khalil Kharrat

**Affiliations:** 1 Department of orthopaedic surgery, Hôtel-Dieu de France Hospital, Saint Joseph University. Beyrouth, Lebanon

## Abstract

**Background:**

Visual estimation (VE) is an essential tool for evaluation of range of motion. Few papers discussed its validity in children orthopedics' practice. The purpose of our study was to assess validity and reliability of VE for passive range of motions (PROMs) of children's lower limbs.

**Methods:**

Fifty typically developing children (100 lower limbs) were examined. Visual estimations for PROMs of hip (flexion, adduction, abduction, internal and external rotations), knee (flexion and popliteal angle) and ankle (dorsiflexion and plantarflexion) were made by a pediatric orthopaedic surgeon (POS) and a 5^th ^year resident in orthopaedics. A last year medical student did goniometric measurements. Three weeks later, same measurements were performed to assess reliability of visual estimation for each examiner.

**Results:**

Visual estimations of the POS were highly reliable for hip flexion, hip rotations and popliteal angle (ρ_c _≥ 0.8). Reliability was good for hip abduction, knee flexion, ankle dorsiflexion and plantarflexion (ρ_c _≥ 0.7) but poor for hip adduction (ρ_c _= 0.5). Reproducibility for all PROMs was verified. Resident's VE showed high reliability (ρ_c _≥ 0.8) for hip flexion and popliteal angle. Good correlation was found for hip rotations and knee flexion (ρ_c _≥ 0.7). Poor results were obtained for ankle PROMs (ρ_c _< 0.6) as well as hip adduction and abduction, the results of which not being reproducible. Influence of experience was clearly demonstrated for PROMs of hip rotations, adduction and abduction as well as ankle plantarflexion.

**Conclusion:**

Accuracy of VE of passive hip flexion and knee PROMs is high regardless of the examiner's experience. Same accuracy can be found for hip rotations and abduction whenever VE is performed by an experienced examiner. Goniometric evaluation is recommended for passive hip adduction and for ankle PROMs.

## Background

Passive range of motion (PROM) measurement is an essential tool for pediatric orthopedists to document disease progression, to plan treatment and to evaluate its results. While many methods are available for ROM evaluation, most physicians consider goniometry as the gold standard and its validity is currently widely accepted especially when measurements are taken by the same examiner within the same session and on the same day [1-16]. Passive ROM measures are generally thought to be more reliable in individuals who have normal tone than in individuals with hypertonicity [11-15]. Kilgour et al [15] found that sagittal plane ROM goniometric measures have similar levels of reliability in children who have spastic diplegia and in control children, both within and between sessions. Validity and reliability of goniometric measurements were well studied in the paper of Gajdosik [16], concluding that the universal full-circle goniometer is the preferred instrument for measuring ROM.

However, in regular daily practice, the majority of clinicians rely on visual estimations (VE) of angles, the reliability of which is yet to be established. In a wide search of the current literature, we found few studies assessing the validity of this method in evaluating the PROM of one or two joints of the lower limb of normal subjects [6,9].

We studied PROM of hips, knees and ankles in a healthy pediatric population.

The aim of this study is threefold:

- To assess the validity of visual estimation

- To study the reproducibility of the results (intra-tester reliability)

- To evaluate the influence of experience (inter-tester reliability)

## Methods

### Subjects

Fifty typically developing children (100 lower limbs) without any orthopedic history were examined. There were 32 girls and 18 boys with an average age of 8 years (3 y-15 y).

### Examiners

The study received ethical approval from our university Ethics Committee and informed consent was given by parents. Three examiners were involved in the study. VE measurements were made by a pediatric orthopaedic surgeon (POS) and a 5^th ^year resident in orthopaedics. A last year medical student did the goniometric measurements.

The specialist is an experienced pediatric orthopedic surgeon (15 years of experience) performing regularly visual estimates for PROMs. The second examiner was a last year resident in orthopedic surgery (5^th ^year of residency), also performing visual estimation in a regular manner. At least one study reporting angular values for joint mobility uses visual estimation of ROMs performed by residents [17]. By comparing values given by the POS to those of the resident, our purpose was to assess the influence of experience. The third examiner who did goniometric measurements was a last year medical student (7^th ^year of medical studies), familiarized with the technique after thorough explanations given by the POS and a practice session. Fish and Wingate [18] have stated that even inexperienced persons can use correctly the goniometer if they know the technique.

### Instrumentation

**Figure 1:**
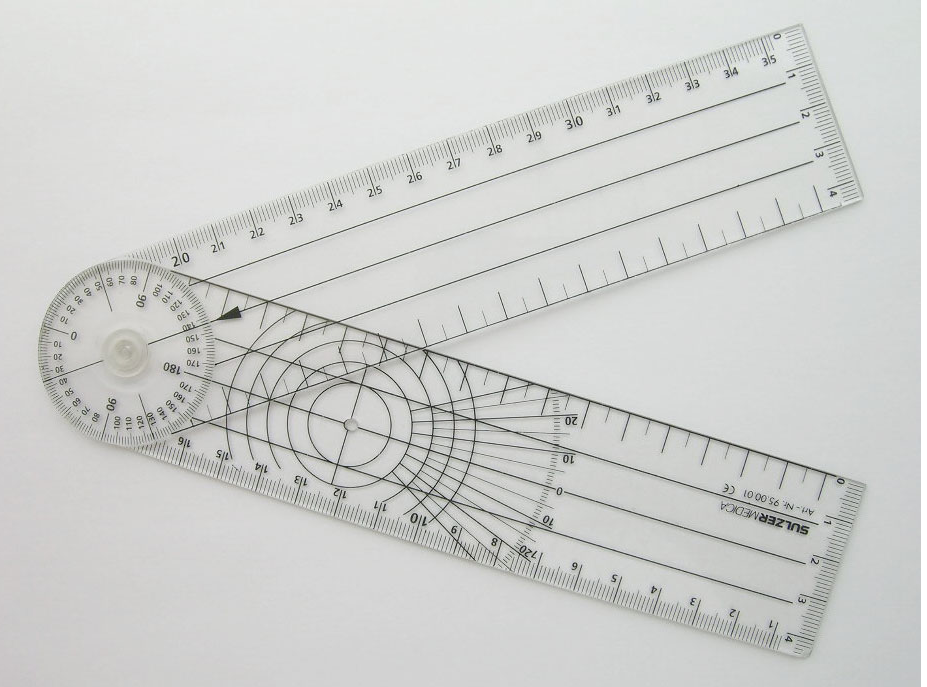
**Two-arm goniometer with a scale marked in 2-degree-increments**.

The measurement tool was the classical two-arm goniometer with a scale marked in 2-degree-increments (figure 1). Validity of the goniometer was verified by measuring ten randomly selected angles drawn by a computer. For visual estimation, PROM measurements were given by multiples of 5 degrees.

### Procedure

The study was divided into two parts.

#### PART 1

All relevant bony landmarks on the lower limbs were exposed and marked on each child (linea alba, middle of inguinal crease as a marker of the center of the hip, greater trochanter, longitudinal axis of the patella, lateral femoral condyle, tibial crest and medial malleolus). We evaluated all PROM measurements in the supine position except for internal and external rotations of the hip (prone position).

Each examiner recorded measurements independently. All motions were passively undertaken and maintained by the pediatric orthopaedic surgeon (POS) at their maximum magnitude, in such a way as to allow visual estimation to be performed for the same amplitude by himself and the resident from the same visual angle, the resident standing right behind the surgeon. Then, and while maintaining the same position, the third examiner did the goniometric measurement to which "visual" examiners were blinded. Standard techniques of goniometric measurements were used with some adjustments to reflect clinical practice [19,20].

For the nine motions described below, the three examiners stood in such a way as to have their visual angle the most perpendicular possible to the plane of motion.

**Figure 2:**
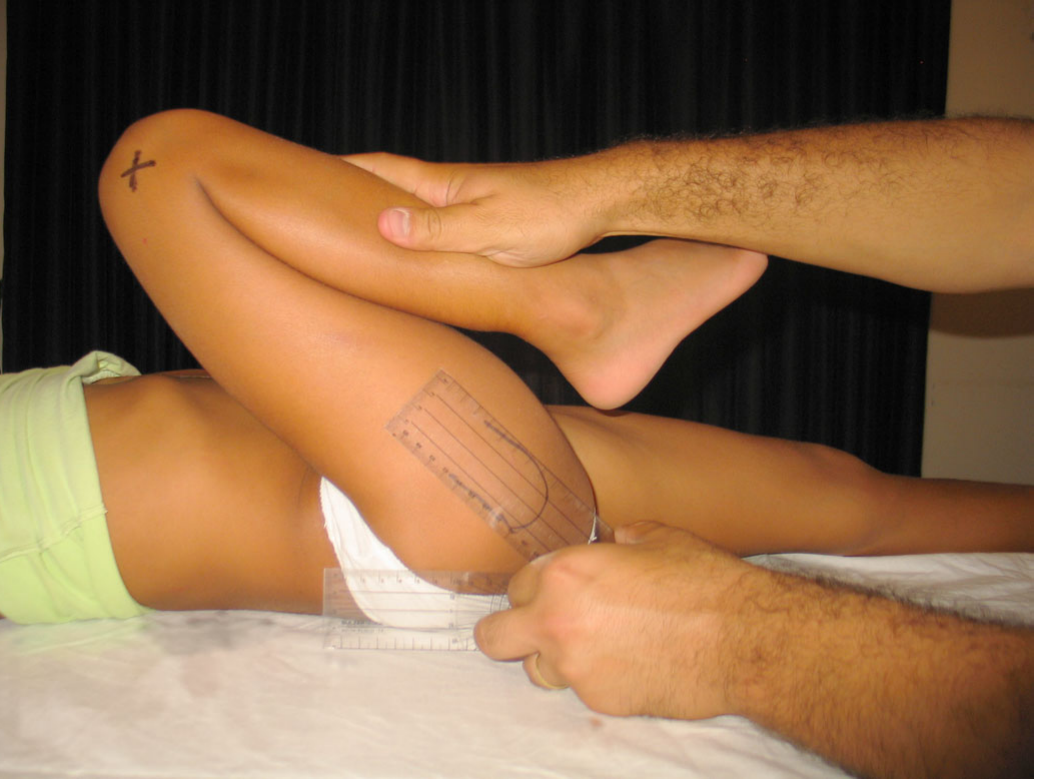
**Hip flexion**.

**Figure 3:**
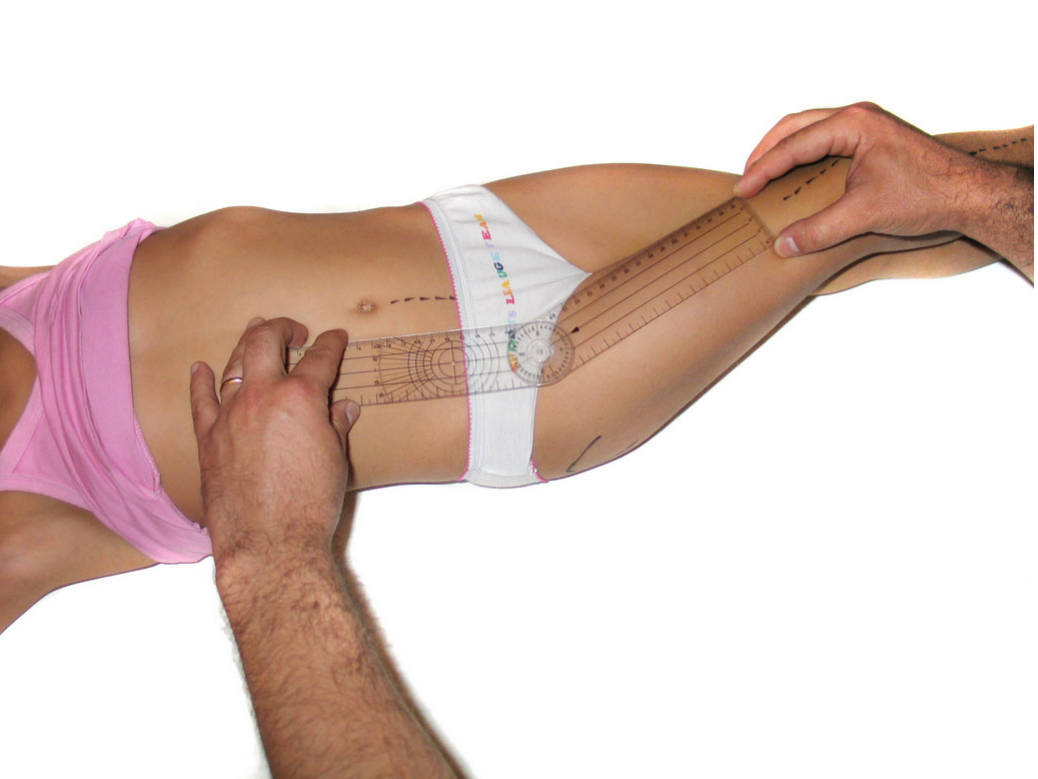
**Hip adduction**.

**Figure 4:**
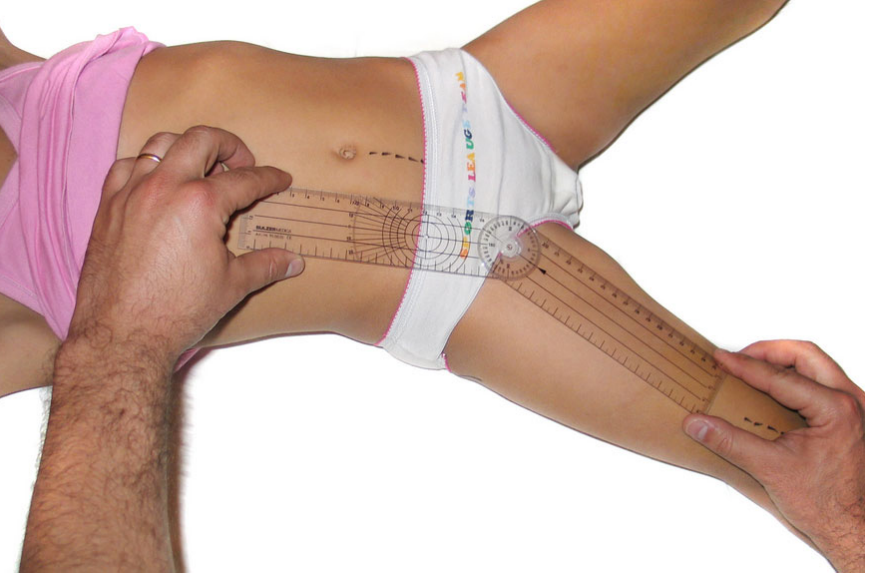
**Hip abduction**.

**Figure 5:**
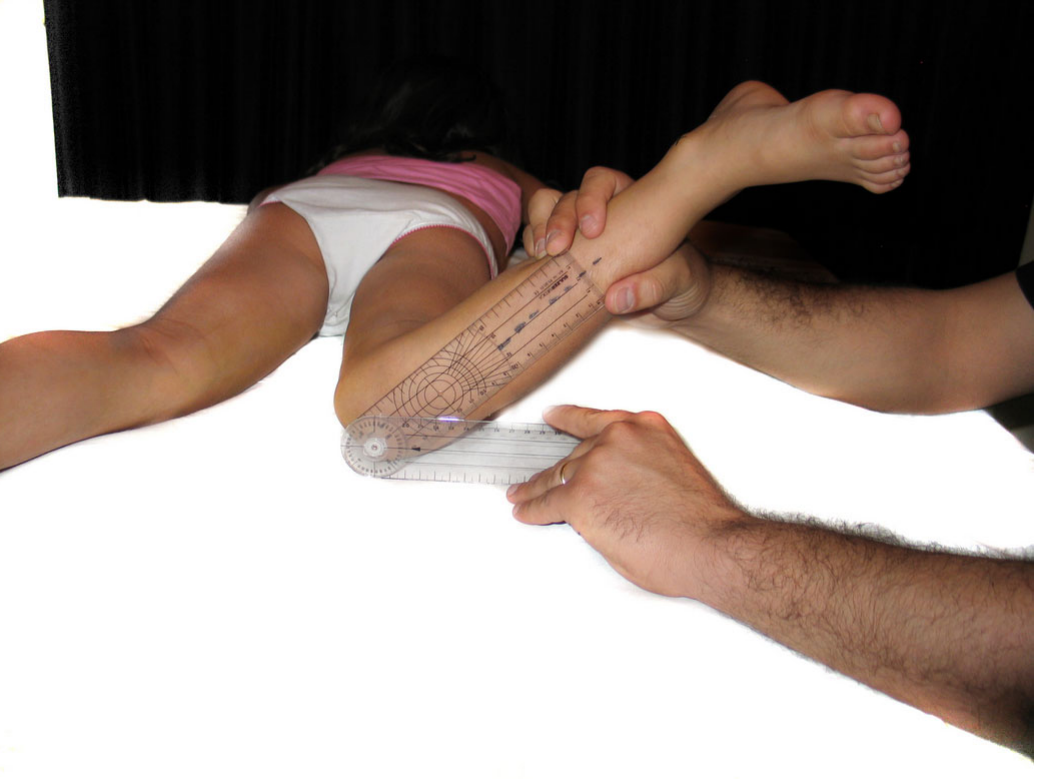
**Hip internal rotation**.

**Figure 6:**
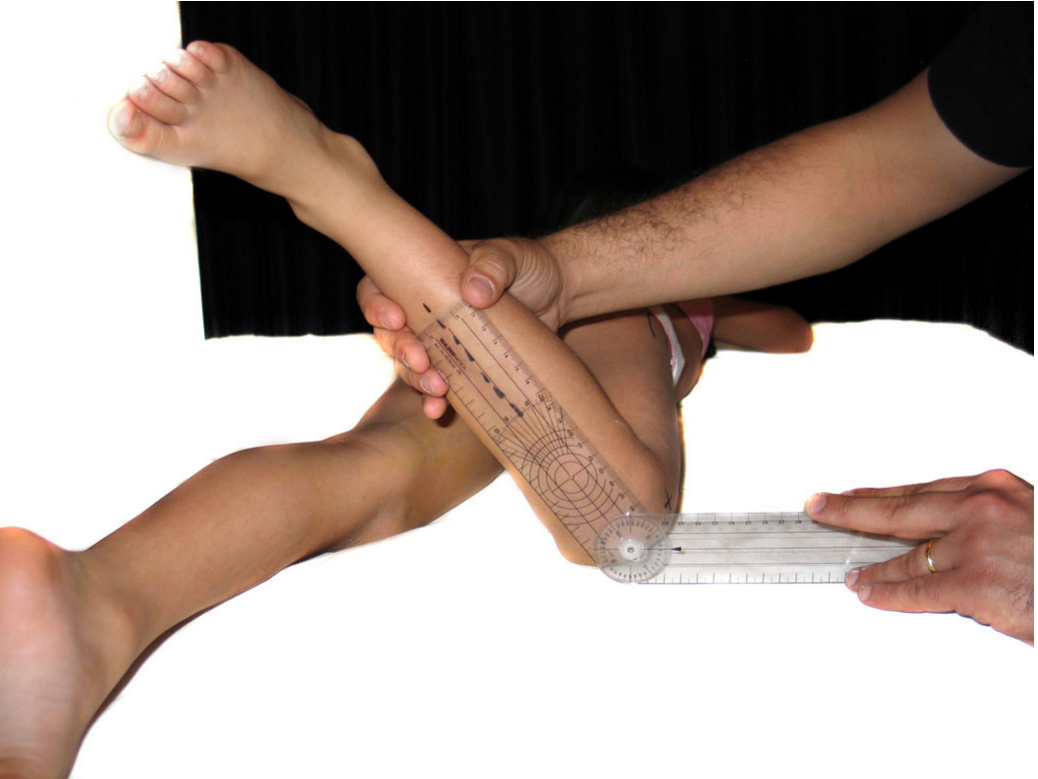
**Hip external rotation**.

**Figure 7:**
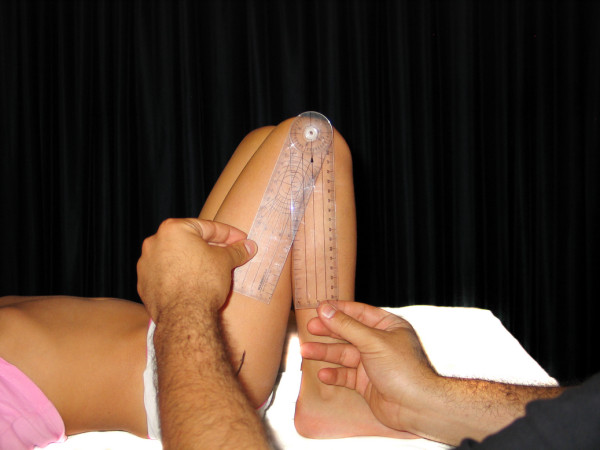
**Knee flexion**.

**Figure 8:**
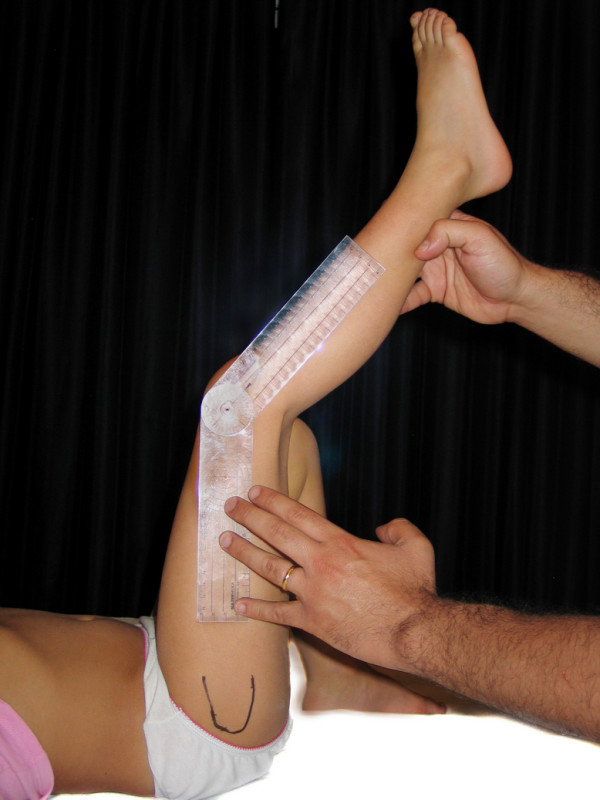
**Popliteal angle**.

**Figure 9:**
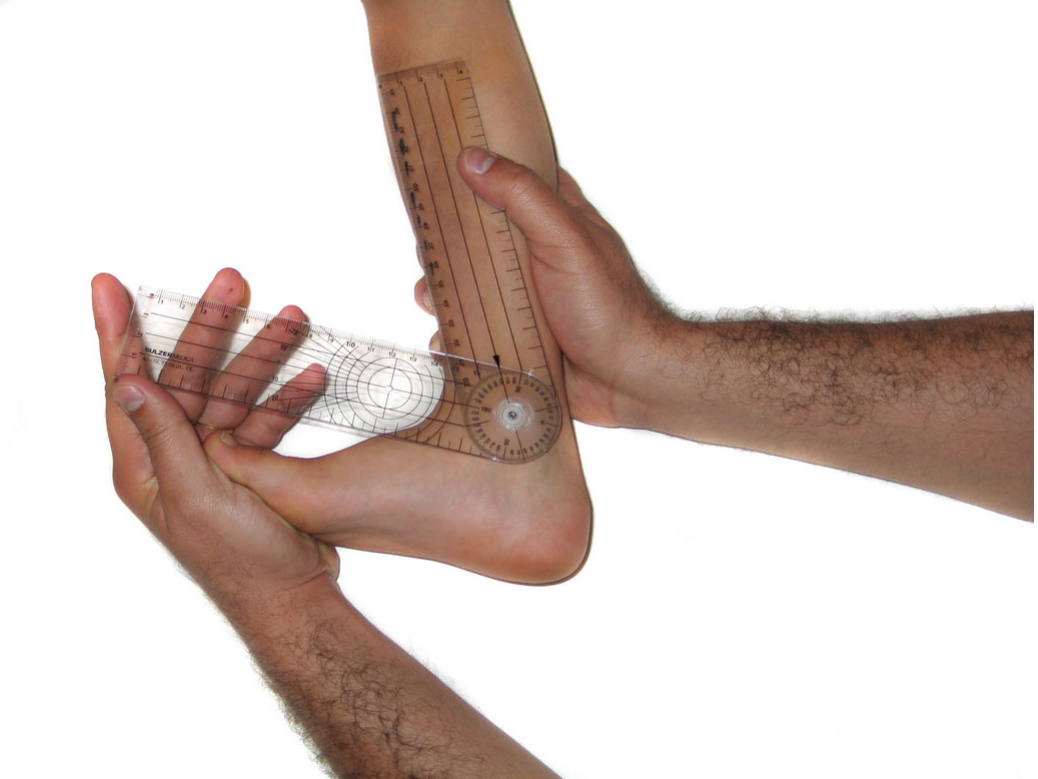
**Ankle dorsiflexion**.

**Figure 10:**
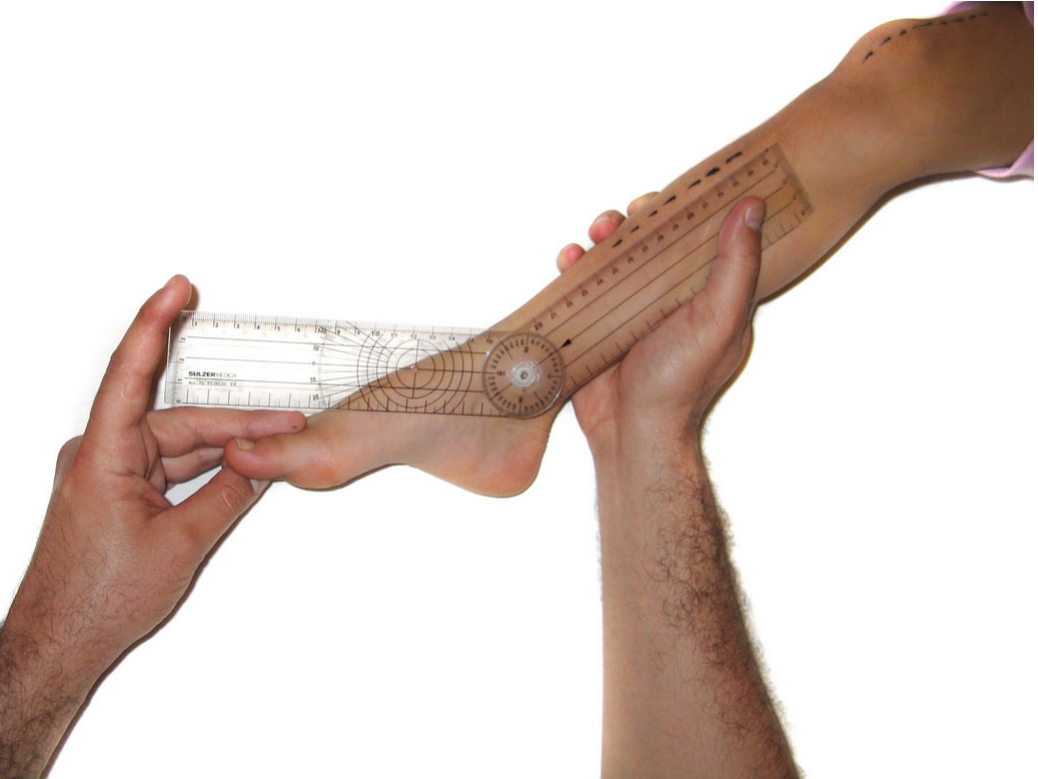
**Ankle plantarflexion**.

- Hip flexion (HFX): The POS flexed the thigh over the abdomen. The student placed one of the goniometer's arms on the examination table and the other along the axis joining the greater trochanter to the lateral femoral condyle (figure 2).

- Hip adduction (HAD): The surgeon adducted the limb over the other one (adduction associated to some degree of flexion). The center of the goniometer was placed over the middle of the inguinal crease, with one of its arms parallel to the linea alba and the other pointing towards the longitudinal axis of the patella (figure 3).

- Hip abduction (HAB): The limb was abducted and the goniometric measurement was performed as described for hip adduction (figure 4).

- Hip internal rotation (HIR): This motion was done in the prone position, with 90 degrees of knee flexion. One of the arms of the goniometer was placed along the examination table and the other in line with the tibial crest (figure 5).

- Hip external rotation (HER): same measurement as for internal rotation but when performing external rotation of the hip (figure 6).

- Knee flexion (KFX): the center of the goniometer was placed over the lateral condyle with one of its arms pointing towards the greater trochanter and the other parallel to the anterior aspect of the leg (figure 7).

- Popliteal angle (PA): Goniometric measurement was performed in the same manner as for knee flexion. We've studied this motion instead of knee extension because the values are more variable and the results are more interesting (figure 8).

- Ankle dorsiflexion (ADF): the foot was dorsiflexed towards the leg. The center of the goniometer was placed over the medial malleolus with one of its arms parallel to the anterior aspect of the leg and the other parallel to the line joining the plantar aspects of the heel and the head of the first metatarsal. We've chosen to examine ROMs of ankle as we usually do in a regular clinical setting which is from the medial side on a patient supine with lower limb externally rotated. Knee was in flexion (figure 9).

- Ankle plantarflexion (APF): same goniometric measurement as for ankle dorsiflexion but when the foot is plantarflexed (figure 10).

#### PART 2

Three weeks later, the same measurements were performed again by the same examiners, on the same children, at the same place, in the same manner and using the same goniometer. Our main objective in this part was to study the reliability of the VE of each examiner. Also, and by studying independently the results of this part, the overall number of measurements is doubled. This part of the study was done three weeks later. Skeletal landmarks were identified again. Some cutaneous markings were still visible from the first part but the majority was erased. We tried to reproduce the same markings by adopting the same techniques of identification of bony landmarks.

### Data analysis

The three methods of measurements (two visual estimations and one goniometric measurement) yield continuous variables. We considered goniometric measurements as the reference to which corresponding visual estimations were compared.

Statistical analysis was done using the test of LIN for calculating the concordance correlation coefficient [21,22] and the graphs of Bland and Altman for measuring the difference between VE and goniometric values for each examiner [23,24].

The test of Lin [21] calculates a concordance correlation coefficient (ccc/rho_c/ρ_c_) for agreement on a continuous measurement obtained by two persons or methods. It evaluates the degree to which pairs of observations fall on the 45° line through the origin. This coefficient ranges from zero (no agreement) to one (perfect agreement). The "ccc" is the product of Pearson correlation coefficient (r) by a bias correction factor (C_b_)



where

• (ρ_c) _is the Lin concordance correlation coefficient; 0 ≤ ρ_c _≤ 1

• (r) is the Pearson correlation coefficient which measures how far each observation deviates from the best-fit line, and is a **measure of precision**; 0 ≤ r ≤ 1

• (C_b) is a bias correction factor that measures how far the best-fit line deviates from the 45° line through the origin, and is a **measure of accuracy**; C_b _< 1

There are no categorized levels for concordance coefficient values but for descriptive reasons we have arbitrarily chosen four categories for correlation: High (ρ_c _≥ 0.8), good (0.7 ≤ ρ_c _< 0.8), fair (0.6 ≤ ρ_c _< 0.7) and poor (ρ_c _< 0.6).

**Figure 11:**
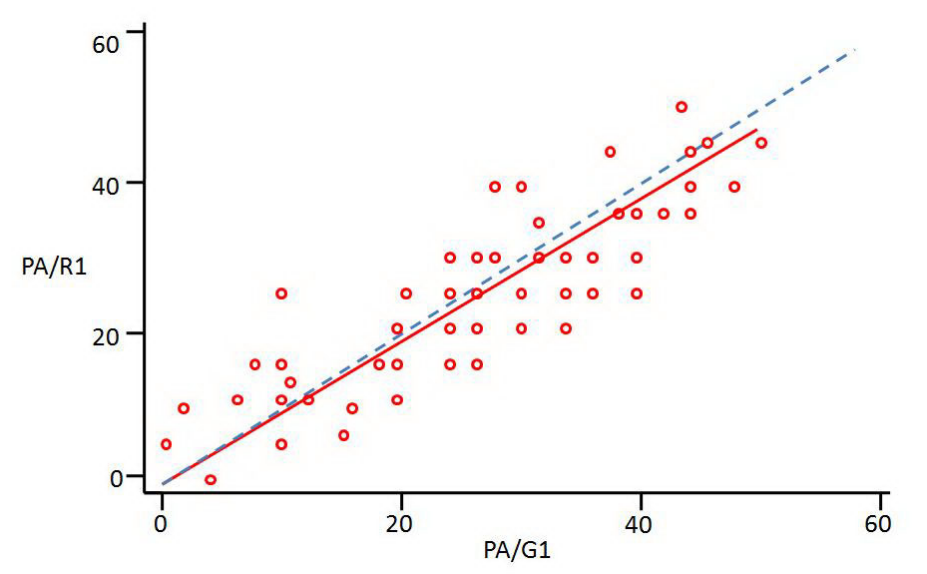
**An example of graphical representation of correlation between visual estimation of popliteal angle (PA) given by the resident (R) and goniometric measurement (G) at the first part of study (1), according to the test of Lin**. Small red circles: pairs of observation; Red line: best-fit line of observations; Dashed blue line: 45 degrees line; measurements are in degrees.

We show in figure 11 an example of graphical representation of correlation between visual estimation of popliteal angle done by the resident and goniometric measurement at the first part of study.

Thus, Lin coefficient combines measures of precision and accuracy. Pearson correlation coefficient is inappropriate for measuring agreement between two methods because it estimates the degree of linear association while ignoring systematic bias. Two methods may have a strong linear association but a poor agreement [23]. Validation of reproducibility was done using the confidence interval approach [25].

Graphs of Bland and Altman are used to graphically represent results obtained by two methods of measurement and are useful to estimate and represent graphically measurement errors. One point is affected for each measure, with the mean value of the two measurements as "x" and their difference as "y". A horizontal line is obtained representing the overall mean difference with two other lines representing limits of agreement at 95% (which means that 95% of the results are between the limits). The overall mean difference line may be over (overestimation) or under (underestimation) the zero line.

**Figure 12:**
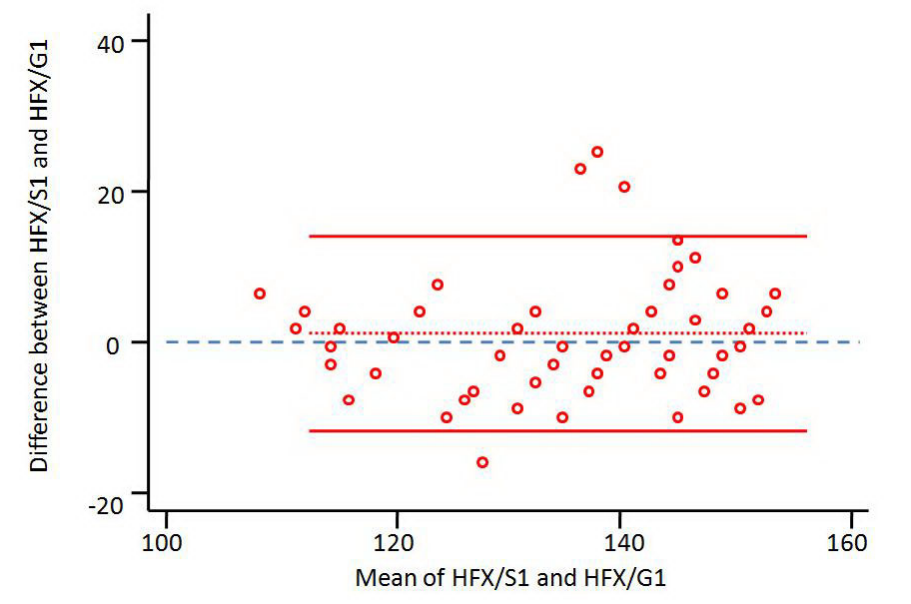
**A representation of Bland-Altman Graph for hip flexion (HFX) estimated by the specialist (S) and compared to goniometric measurements (G) in the first part of the study (1)**. Small red circles: representations for couples of observation with mean value of measurements as "x" and their difference as "y"; Dotted red line: mean measurement error; Solid red lines: limits of agreement at 95%; Blue dashed line: zero line; measurements are in degrees.

We show in figure 12 a graphical representation of specialist's estimations for hip flexion compared to goniometric measurements, according to Bland-Altman method.

We reached the three main objectives of our study by:

- Assessing the concordance correlation coefficient [21] and the graphs of Bland-Altman [23] (study of validity of visual estimation)

- Comparing respective values of "ccc" obtained in the two parts of study for each examiner seperately by using the confidence interval method [25] (intra-rater reliability). We've deliberately chosen this method of evaluation of intra-rater reliability to avoid bias related to direct comparison of visual estimations obtained in parts 1 and 2 of the study. PROMs are very difficult to reproduce and respective ROMs obtained in the two parts of the study are certainly different [8,9,15]. This problem is thoroughly explained in the discussion section.

- Comparing corresponding values of "ccc" for the two examiners within each part of the study by using the confidence interval method [25] (inter-rater reliability and influence of experience). This method demonstrates whether a measurement tool is reliable or not, without calculating a coefficient.

## Results

### 1. Assessment of validity of visual estimation

#### 1.1 Pediatric orthopaedic surgeon

Correlation between visual estimations (VE) of the POS and goniometric measurements for the two parts of study is summarized in [see Additional file 1 Stable 1]. We found high reliability for hip flexion, hip rotations and popliteal angle (ρ_c _≥ 0.8). Reliability was good for hip abduction, knee flexion, ankle dorsiflexion and plantarflexion (ρ_c _≥ 0.7). Concordance was poor for hip adduction (ρ_c _= 0.5). In all cases, correlation was statistically significant (p < 0.001).

We present in [see Additional file 2 Stable 2] a simplified representation of Bland-Altman graphs for the nine PROMs for all measurements of the study (parts 1 and 2) showing mean measurement errors of visual estimation (in degrees) when compared to goniometric measurements. We show 95% confidence interval as well. There is a general tendency to overestimate hip flexion, hip abduction and ankle plantarflexion. Knee PROMs and ankle dorsiflexion are underestimated. Correlation was statistically significant in all cases (p < 0.001).

#### 1.2 Resident

The accuracy of the resident's VE for the two parts of study is presented in [see Additional file 3 Stable 3]. Hip flexion and popliteal angle showed high reliability (ρ_c _≥ 0.8). The correlation was good for hip rotations and knee flexion (ρ_c _≥ 0.7). Results were poor for hip adduction and abduction and for ankle PROMs (ρ_c _< 0.6).

In [see Additional file 4 Stable 4], we show mean measurement errors in degrees with 95% confidence interval according to the graphs of Bland-Altman for all measurements. There is a tendency to overestimation for hip flexion and adduction. Hip abduction, hip internal rotation, knee PROMs and ankle dorsiflexion are generally underestimated.

### 2. Study of reproducibility (intra-tester reliability)

#### 2.1. Pediatric orthopaedic surgeon

By using the confidence interval method of respective concordance correlation coefficient, we demonstrated reproducibility for all PROMs.

#### 2.2. Resident

We demonstrated lack of reproducibility for hip adduction and abduction while visual estimations of other PROMs were found to be reliable.

### 3. Evaluation of the role of experience (inter-tester reliability)

As visual estimations given by the resident are not reliable for hip adduction and abduction, we deduce the importance of level of experience for these PROMs. The role of experience was also demonstrated for hip rotations and ankle plantarflexion using the confidence interval approach.

## Discussion

This study brings up the advantages and limitations of visual estimation of PROMs in the pediatric lower limb.

This is to our knowledge the first study evaluating reliability of VE for hip PROMs. We found high level of accuracy of VE for hip flexion and rotations, and good accuracy for hip abduction, but a lack of reproducibility of measurements performed by a less experienced examiner. Concordance was poor for hip adduction (ρ_c _≈ 0.5). Many factors may have contributed to these poor results of VE of hip adduction: the examination technique with the hip flexed and the low absolute values of hip adduction compared to flexion, abduction and rotations. The level of experience was found to be important in estimating hip PROMs other than flexion.

Marks et al [26] investigated the reliability of VE of knee ROM taken by three physicians on patients with rheumatoid arthritis, who examined the patients independently. One could assume that each examiner may have applied a different force to move the joint, theoretically leading to bias in interpretation of VE. Despite this possible bias, the authors found good intra- and interexaminer reliability. In order to avoid a similar bias in our study, PROMs for each joint were performed once by the same examiner.

Watkins et al [10] studied passive flexion and extension of 50 knees. For each tested knee, two physical therapists were chosen randomly from a list of 14 examiners. In this study as well, estimations were done separately by each examiner in a position of his choice. Visual estimations were performed before goniometric measurements for each joint. We believe that this may influence subsequent visual assessment by adjustment of visual estimations. In our study, the two examiners who did visual estimation never knew goniometric values of PROMs. Although the ICC (Intraclass Correlation Coefficient used by Watkins et al [10] is less reliable than the CCC (Concordance Correlation Coefficient) [21] used in our study, we found similar good concordance for knee flexion and high concordance for popliteal angle. Therefore, our results are similar to those reported by previous studies [10,27] which found good accuracy for visual estimation of knee PROMs, with a tendency to slightly underestimate real values. Experience does not seem to play a major role in VE of knee PROMs. Even unexperienced examiners may satisfactorily estimate knee motions without using a goniometer.

Youdas et al [7] evaluated active range of motion (AROM) of 45 ankles (dorsiflexion and plantar flexion). The study included 10 physical therapists who performed measurements with their own preferences regarding position and method. They assessed interobserver reliability for visual estimation and intraobserver reliability for visual estimation and goniometry. AROMs are much more subject to variations than PROMs. The authors found low concordance between VE of different examiners (ICC = 0,34 for dorsiflexion and ICC = 0,48 for plantarflexion) and a fair concordance between visual estimation and goniometry (0,58 for dorsiflexion and 0,625 for plantarflexion). We believe that these low values are mainly due to the fact that AROMs are much more subject to variations than PROMs and to the absence of standardization of goniometric measurements as recognized by the authors themselves.

Allington et al [2] assessed intra- and interobserver reliability and reproducibility of goniometry and visual estimation of ankle PROMs in 24 children with spastic cerebral palsy (46 ankles). Two physical therapists performed all the measurements. They found very good correlations between the goniometer and the naked eye (r > 0,94) for ankle dorsiflexion and plantarflexion. They also found a mean error (ME) of 5° with a SD of 5° in the inter- and intraobserver measurements. Thus, even with very good reliability, there is a significant error to be taken into account when performing visual estimates. In our study, we found good level of concordance between the specialist's VE and goniometry for ankle PROMs (ρ_c _≈ 0.7). We do not have an explanation for the very high concordance observed in the study of Allington et al [2] but we believe that this may be partly due to the small number of patients examined.

Disparity in results of VE of ankle PROMs between the different studies is due to many factors: the number of examined ankles, the maneuver technique and the method of goniometric measurement.

One of the major limitations of our study was the small number of examiners. It is obvious that by increasing their number with different levels of experience, more conclusions could be stated concerning the role of experience. Even though there were only two examiners performing visual estimation, we believe that results can be extrapolated to other experienced and less experienced examiners.

Validity and reliability of goniometric measurements were not verified in our study. This could have been done for example by an additional examiner doing another set of goniometric measurements, but we really think that this problem was thoroughly discussed and verified in the literature [1-16] and we've deliberately chosen goniometric measurements as a reference to study more precisely visual estimation and the role of experience. Considering all measurements as variables to be verified would have certainly complicated the statistical analysis and weakened the conclusions. Based on literature statements and on our standardized technique for goniometric measurements, we can assume with some caution that these measurements were valid and reliable.

While performing the examination, we sometimes encountered lack of compliance especially with younger children, making measurements difficult to obtain. In addition, the force applied on the limb may vary for the same range of motion not only from one child to another, but also for the same child when repeated measurements are done. Different results could have been then obtained if the same motion was repeated by each examiner. This problem was discussed by Amis and Miller [28] who explained that passive motions are difficult to reproduce because stretching of soft tissues at the limit of the motion depends on the force applied on the limb. Wagner [29] found a greater variability when measuring passively motions of pronation and supination of the forearm. Kilgour (15) demonstrated that while some measurement error arises during the placement and reading of the goniometer, the majority of it is most likely related to difficulties in determining end-range joint positioning. Perhaps force dynamometers (including those that are hand-held) could be used to standardize the amount of passive force applied and thereby decrease the potential for error [30]. We tried to limit the bias relative to these problems by excluding children of three years or less, and by maintaining the same position while doing the three measurements (two visual estimations and one goniometric measurement). But while this may enhance reliability by reducing error, it does not reflect clinical practice where examiners perform ROMs separately. For this reason, we think that a patient should be followed by the same therapist to document disease progression or to evaluate treatment results.

While comparing the charts of measurements (visual and goniometric) of the two parts of our study, we noticed that for a given child, respective values of PROMs are different and this difference is sometimes up to 20 degrees for both measurements (visual and goniometric). This can be hardly explained by the sole visual or goniometric error and we are sure that a large part of variation is due to change in PROM itself. To avoid such an important bias, we tested intra-rater reliability by comparing respective concordance coefficients instead of direct comparison of visual estimations. We can also understand in this setting why it is misleading to study the reliability of goniometric measurements by comparing the two sets of measurements.

We had some difficulties with goniometric measurements. Short limbs were easier to measure because landmarks were closer to the goniometer's arms. This was especially true for motions around the hip and the knee. In retrospect, a longer armed goniometer would have decreased some of the problems. On the other hand, some bony landmarks were moving under the skin. Therefore, the greater trochanter was marked while flexing the hip to minimize variations.

We had some overweight children. Obesity can make estimations difficult to obtain. It may also hide some bony landmarks especially the greater trochanter. This is added to the fact that, naturally, identification of bony landmarks is more difficult in the lower limb [31]. The AAOS [32] and Rowe [27] have suggested that visual estimation is more reliable than goniometric measurements when bony landmarks are not easily seen or palpated. However, overweight children were not excluded from our study to avoid the bias of selection.

## Conclusion

Visual estimation (VE) of passive hip flexion, knee flexion and popliteal angle is highly accurate and reliable regardless of the examiner's experience. VE of hip rotations and abduction is also highly accurate provided it is performed by an experienced examiner. Goniometric evaluation is preferred for passive hip adduction especially if it is performed by less experienced examiners. Inexperienced testers should not estimate hip adduction and abduction for their measurements are not reliable for these PROMs. Goniometry has to be used in such situations. The ankle seems to be the joint with the least reliability for VE of PROMs, and this should be kept in mind and taken into consideration when reporting treatment results in scientific papers.

## Competing interests

The authors declare that they have no competing interests.

## Authors' contributions

IG conceived of the study. RR, IG and IK performed the study and participated in its design. SEH participated in the design of the study and in the statistical analysis. KK and FD reviewed the paper and made corrections. All authors read and approved the final manuscript.

## Pre-publication history

The pre-publication history for this paper can be accessed here:



## Supplementary Material

Additional file 1 **Correlation between specialist's VE and goniometric measurements**. Abscissa axis represents the different PROMs; ordinate axis represents values of the concordance correlation coefficient (ρ_c_). *HFX: hip flexion, HAD: hip adduction, HAB: hip abduction, HIR: hip internal rotation, HER: hip external rotation, KFX: knee flexion, PA: popliteal angle, ADF: ankle dorsiflexion, APF: ankle plantarflexion; 1(blue columns) and 2(red columns) correspond to the two parts of the study*. Click here for file

Additional file 2 **Mean measurement error for all PROMs estimated by the specialist**. Short horizontal lines represent mean error measurement in degrees; vertical lines represent confidence interval at 95%. *HFX: hip flexion, HAD: hip adduction, HAB: hip abduction, HIR: hip internal rotation, HER: hip external rotation, KFX: knee flexion, PA: popliteal angle, ADF: ankle dorsiflexion, APF: ankle plantarflexion*. Click here for file

Additional file 3 **Correlation between Resident's VE and Goniometric measurements**. Abscissa axis represents the different PROMs; ordinate axis represents values of the concordance correlation coefficient (ρ_c_). *HFX: hip flexion, HAD: hip adduction, HAB: hip abduction, HIR: hip internal rotation, HER: hip external rotation, KFX: knee flexion, PA: popliteal angle, ADF: ankle dorsiflexion, APF: ankle plantarflexion; 1(blue columns) and 2(red columns) correspond to the two parts of the study*. Click here for file

Additional file 4 **Mean measurement error for all PROMs estimated by the resident**. Short horizontal lines represent mean error measurement in degrees; vertical lines represent confidence interval at 95%. *HFX: hip flexion, HAD: hip adduction, HAB: hip abduction, HIR: hip internal rotation, HER: hip external rotation, KFX: knee flexion, PA: popliteal angle, ADF: ankle dorsiflexion, APF: ankle plantarflexion*. Click here for file
