# Supplementary figures and images for: Is visual estimation of passive range of motion in the pediatric lower limb valid and reliable
Source: BMC Musculoskelet Disord. 2009 Oct 12;10:126. doi: 10.1186/1471-2474-10-126 (PMC2765954; doi:10.1186/1471-2474-10-126)

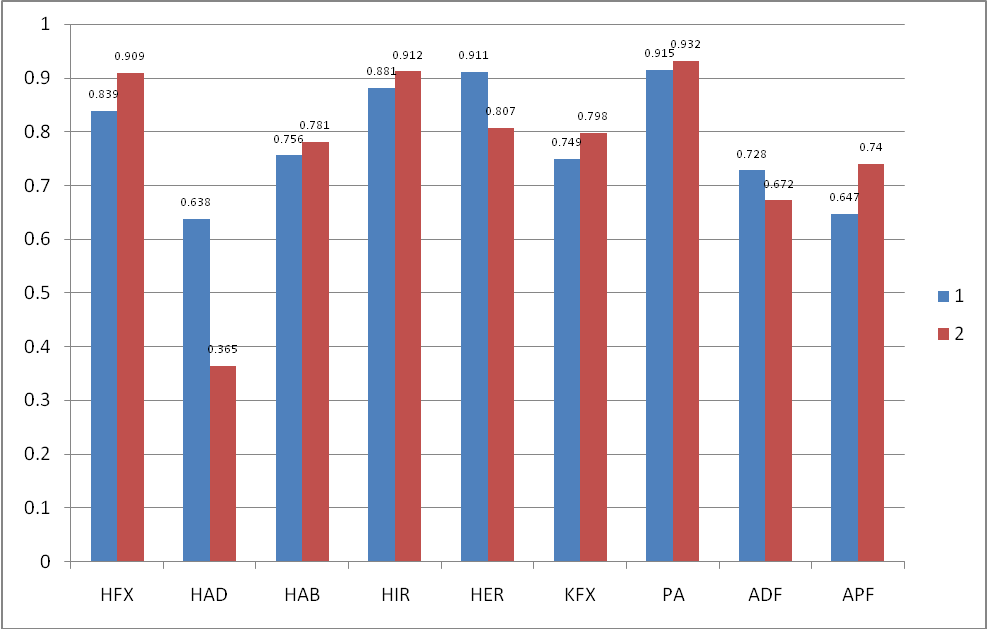

Supplement: Additional file 1 — Correlation between specialist's VE and goniometric measurements. Abscissa axis represents the different PROMs; ordinate axis represents values of the concordance correlation coefficient (ρc). HFX: hip flexion, HAD: hip adduction, HAB: hip abduction, HIR: hip internal rotation, HER: hip external rotation, KFX: knee flexion, PA: popliteal angle, ADF: ankle dorsiflexion, APF: ankle plantarflexion; 1(blue columns) and 2(red columns) correspond to the two parts of the study. [file 1471-2474-10-126-S1.DOC]

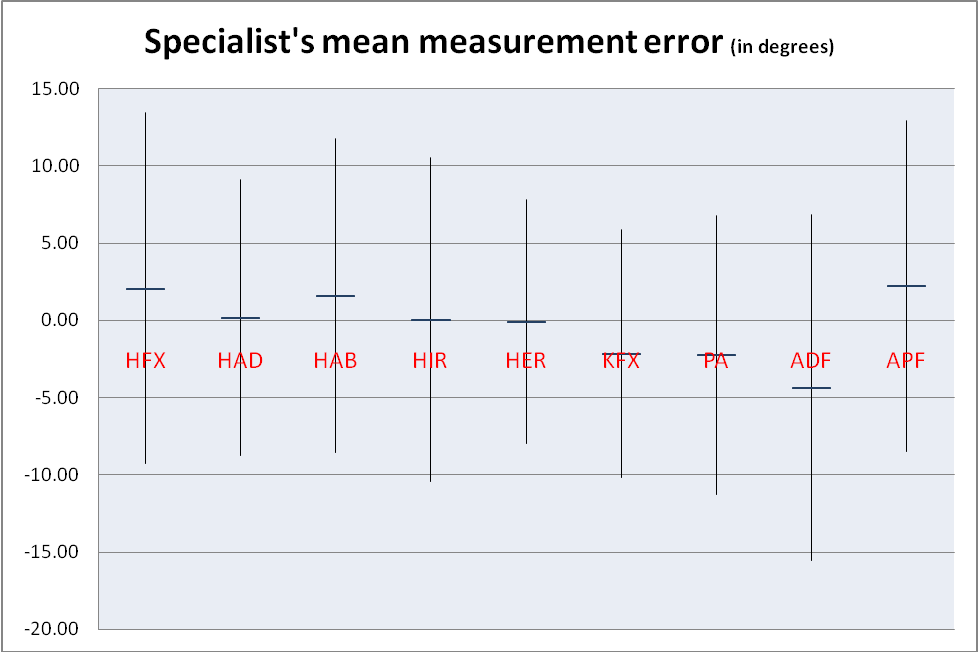

Supplement: Additional file 2 — Mean measurement error for all PROMs estimated by the specialist. Short horizontal lines represent mean error measurement in degrees; vertical lines represent confidence interval at 95%. HFX: hip flexion, HAD: hip adduction, HAB: hip abduction, HIR: hip internal rotation, HER: hip external rotation, KFX: knee flexion, PA: popliteal angle, ADF: ankle dorsiflexion, APF: ankle plantarflexion. [file 1471-2474-10-126-S2.DOC]

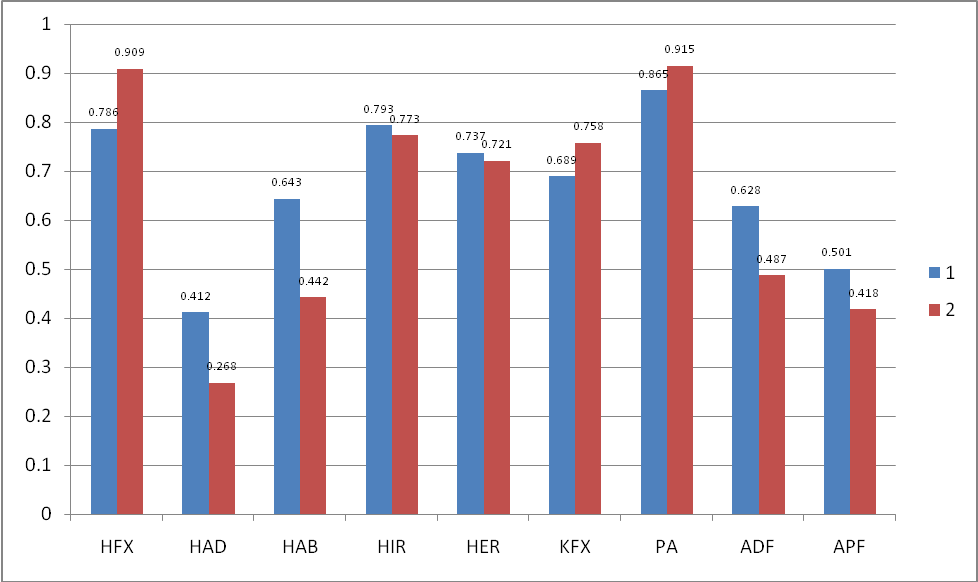

Supplement: Additional file 3 — Correlation between Resident's VE and Goniometric measurements. Abscissa axis represents the different PROMs; ordinate axis represents values of the concordance correlation coefficient (ρc). HFX: hip flexion, HAD: hip adduction, HAB: hip abduction, HIR: hip internal rotation, HER: hip external rotation, KFX: knee flexion, PA: popliteal angle, ADF: ankle dorsiflexion, APF: ankle plantarflexion; 1(blue columns) and 2(red columns) correspond to the two parts of the study. [file 1471-2474-10-126-S3.DOC]

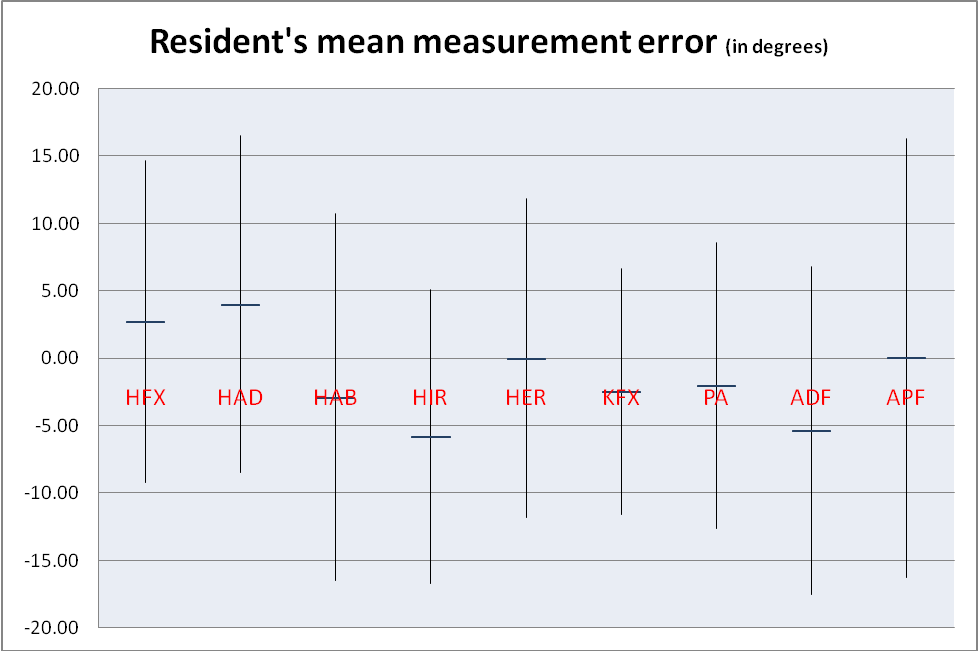

Supplement: Additional file 4 — Mean measurement error for all PROMs estimated by the resident. Short horizontal lines represent mean error measurement in degrees; vertical lines represent confidence interval at 95%. HFX: hip flexion, HAD: hip adduction, HAB: hip abduction, HIR: hip internal rotation, HER: hip external rotation, KFX: knee flexion, PA: popliteal angle, ADF: ankle dorsiflexion, APF: ankle plantarflexion. [file 1471-2474-10-126-S4.DOC]
